# Resin Wing Technique With Sandblasting Interface Modification: A 24-Month Stable Pulp Preservation in Young Permanent Teeth With Complex Crown Fractures

**DOI:** 10.1155/crid/4504857

**Published:** 2025-10-01

**Authors:** Jian Zhang

**Affiliations:** Department of Endodontics, The Affiliated Stomatological Hospital of Jiujiang University, Jiujiang, Jiangxi, China

**Keywords:** complex crown fracture, dental trauma, fragment reattachment, pulp preservation, resin wing

## Abstract

**Background:**

Dental trauma, especially crown fractures of the maxillary anterior teeth, has a very high incidence. Conventional methods for repairing crown fractures of anterior teeth include direct resin restoration, veneers, crown restoration, and crown fragment reattachment. This case report details the treatment process of pulp preservation and the use of resin wing technique for crown fragment reattachment in a young patient with a secondary complex crown fracture of the maxillary anterior teeth.

**Case Description:**

In this case report, the author detailed the treatment process of a young patient with secondary complex crown fractures on the upper anterior teeth. During the first treatment for crown fracture, pulpotomy and crown fragment reattachment were performed on the damaged tooth. In the second treatment for crown fracture, the bonding interface was roughened using sandblasting technology, and the fractured tooth fragment was precisely reattached using the resin wing method. After 24 months of follow-up observation, the treatment effect remained stable.

**Conclusion:**

Pulp preservation combined with fragment reattachment represents an optimal approach for managing complex crown fractures in young permanent teeth. The resin wing technique improved repositioning precision and reduced technical sensitivity, enabling efficient case completion. Nevertheless, case selection—considering factors such as functional support of adjacent teeth—is critical for technique applicability.

Dental trauma is highly prevalent, with a particular propensity for crown fractures in the maxillary anterior teeth. The primary restorative approaches for anterior crown fractures include direct resin restoration, veneers, crown restoration, and fragment reattachment [1–4]. When a fractured tooth segment is salvageable, adhering to the guidelines of the International Association of Dental Traumatology (IADT) from 2020, the use of adhesives to reattach the fractured segment is the preferred clinical method for managing dental trauma involving crown fractures [5–8]. This technique is capable of rapidly restoring the original tooth's shape, color, translucency, and surface texture, also achieving both esthetics and functionality [7, 9, 10]. Currently, achieving a perfect match at the bonding interface and precise repositioning of the fractured segment in complex crown fractures remains challenging.

The author attempted to treat a patient with a complex crown fracture of the maxillary anterior teeth on two occasions. During the initial crown fracture treatment, a pulpotomy and fragment reattachment technique were performed on the affected tooth. In the subsequent treatment for crown fracture, the affected tooth was sandblasted to roughen the bonding interface, and a resin wing technique was employed for the reattachment of the fractured segment. This approach restored the original morphological characteristics of the tooth and preserved the vitality of the pulp. After a follow-up period of 24 months, the results were favorable. The case is reported as follows.

## 1. Case Presentation

### 1.1. Patient Information

#### 1.1.1. General Condition

The patient is a 12-year-old male who presented to the Affiliated Stomatological Hospital of Jiujiang University in February 2023, complaining of “trauma to the upper right anterior tooth for 4 h.” The patient had accidentally fallen 4 h prior, resulting in a fracture of the crown of the upper right anterior tooth. There is no history of systemic diseases, family genetic diseases, or allergies.

#### 1.1.2. Clinical Examination

**Figure 1:**
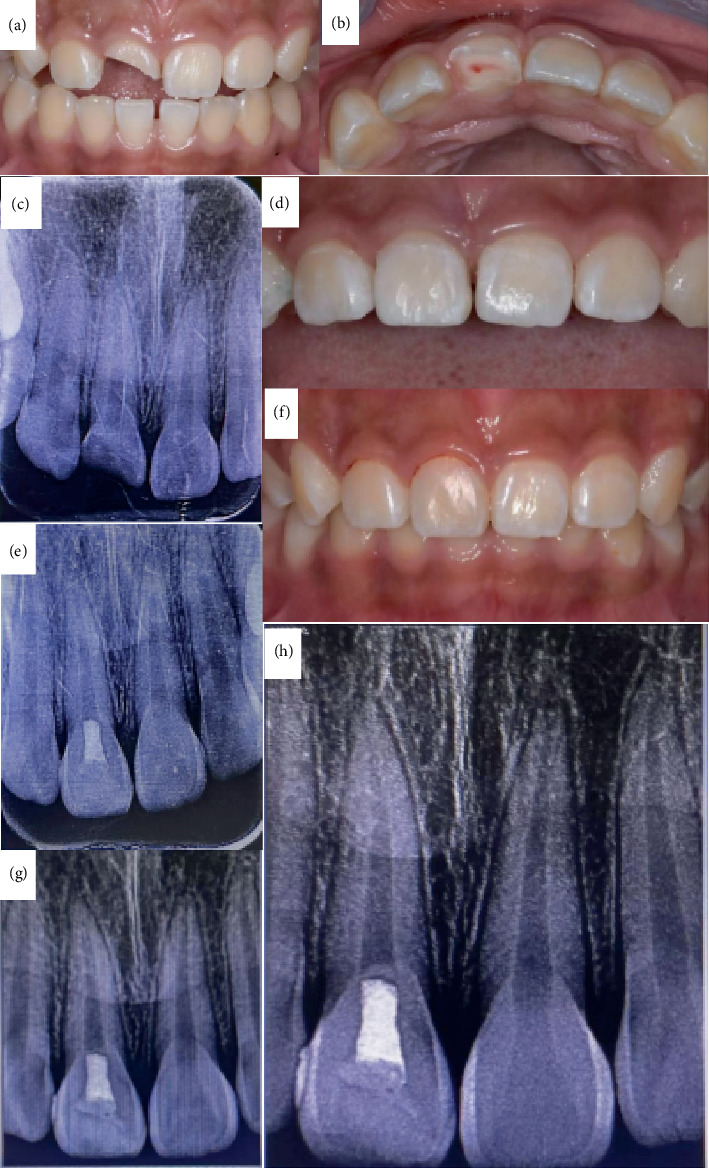
Patient treatment process. (a) Initial frontal view shows fracture of the crown of Tooth 11. (b) Initial incisal view shows exposure of the pulp hole at the fracture end of Tooth 11. (c) Initial periapical radiograph shows fracture of the crown of Tooth 11, root development at Nolla Stage 9, no image of root fracture, continuous periodontal ligament without widening. (d) Immediately after pulpotomy and crown reattachment surgery for Tooth 11. (e) Periapical radiograph immediately after surgery for Tooth 11. (f) Frontal view at 1-month follow-up after crown reattachment surgery. (g) Periapical radiograph at 1-month follow-up after crown reattachment surgery shows slight calcification bridge image change at the root canal orifice of Tooth 11, with no abnormalities seen in the periapical area. (h) Periapical radiograph at 6-month follow-up after crown reattachment surgery shows significant calcification bridge image change at the root canal orifice of Tooth 11, with no abnormalities seen in the periapical area.

The patient exhibits a symmetrical facial shape, with fair oral hygiene and no soft plaque. The mouth opening and occlusion are normal, and there is no clicking or pain in the temporomandibular joint. Tooth 11 has an oblique fracture extending from mesial to distal by two-thirds, with a lingual defect that extends 0.2 mm below the gumline. The fracture end is red, with an exposed pulp chamber approximately 1.0 mm in diameter. The tooth is sensitive to probing (++) and has no mobility. The gingiva appears normal in color, shape, and texture, and no deep periodontal pockets are detected. Tooth 12 has an intact crown, with no response to probing (−), no percussion pain (−), and no mobility. Tooth 21 also has an intact crown, with no response to probing (−), no percussion pain (−), and no mobility. On maximum intercuspation, the anterior teeth exhibit deep overbite, and no significant abnormalities are observed in the remaining teeth. (Figure 1a,b).

#### 1.1.3. Radiographic Examination

The periapical radiograph indicates that Tooth 11 has a crown fracture, the root development is at Nolla Stage 9, the root canal walls are continuous, and the periodontal ligament is continuous without widening; Tooth 21 has continuous root canal walls, with root development at Nolla Stage 9 (Figure 1c).

### 1.2. Diagnosis

The diagnosis is traumatic crown fracture of Tooth 11.

### 1.3. Treatment Plan

1. Attempt pulpotomy and crown reattachment for Tooth 11.
2. Suggest permanent restoration of Tooth 11 after reaching adulthood.
3. Regular follow-up.

### 1.4. Treatment Process

Prior to the commencement of treatment, the patient and their family were thoroughly briefed regarding the medical condition, the proposed treatment regimen, and the associated expenses. Subsequently, they provided their informed consent by signing the requisite documentation.

**Figure 2:**
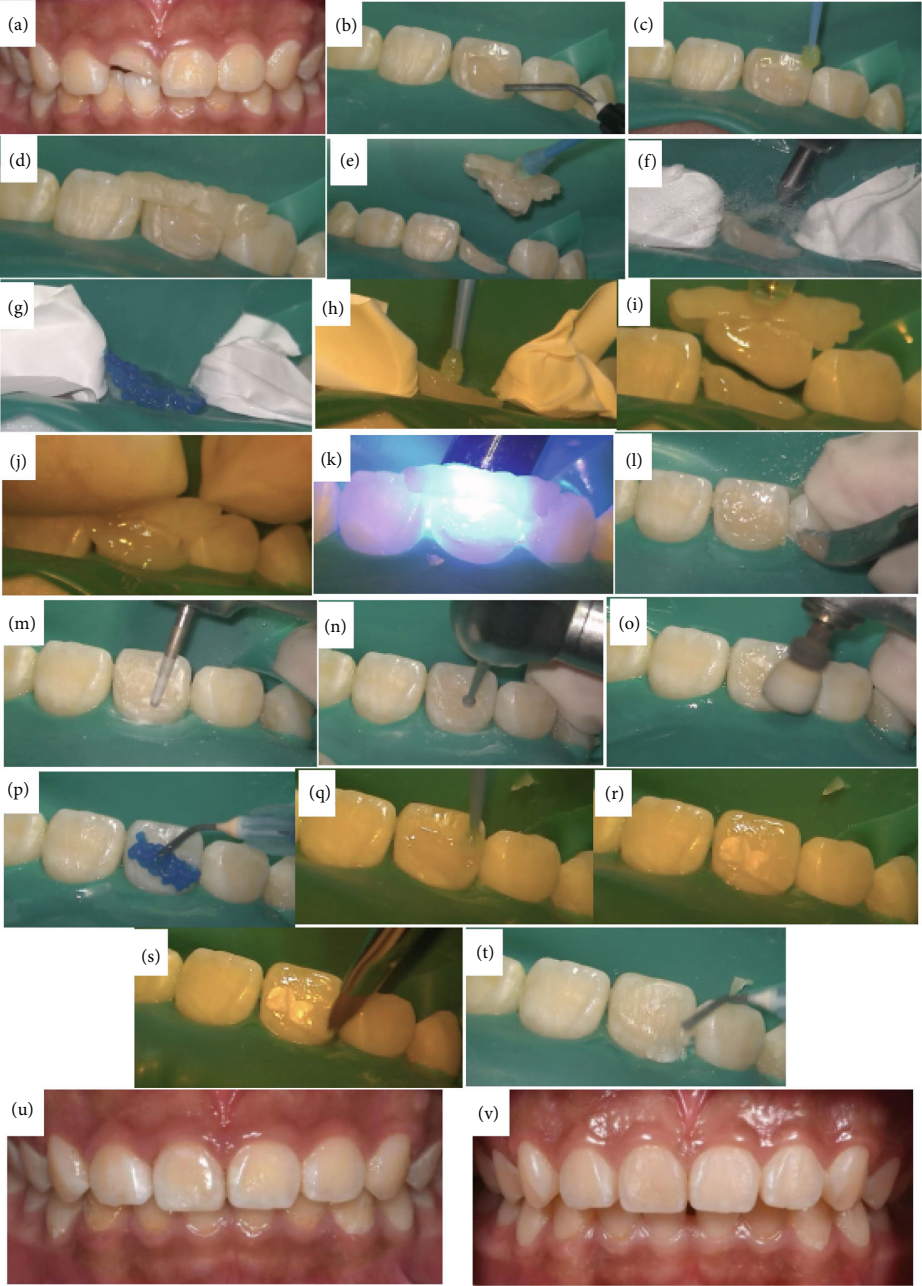
Patient treatment process. (a) Frontal view of the second crown fracture of Tooth 11. (b) Preliminary fixation of the fractured tooth segment with flowable resin. (c) Application of eighth-generation self-etch adhesive without light curing at the incisal end. (d) Coverage of the incisal end of Tooth 11 and the incisal angles of adjacent teeth with composite resin. (e) Completion of the resin wing fabrication. (f) Aluminum oxide sandblasting of the bonding interface at 50 *μ*m. (g) Etching with 35%phosphoric acid. (h) Application of eighth-generation self-etch adhesive. (i) Bonding of the fractured crown with F00 flowable resin. (j) Complete seating of the fractured tooth segment combined with the resin wing. (k) Light curing after removing excess resin. (l) Removal of residual resin from the proximal surface with a 12D blade. (m) Removal of labial resin with a diamond bur. (n) Preparation of a shallow concave bevel at the labial fracture line. (o) Polishing with silicon particle. (p) Etching. (q) Application of adhesive. (r) Composite resin restoration. (s) Shaping with a brush. (t) Application of oxygen inhibitor and light curing. (u) Immediate postoperative frontal view. (v) Frontal view at the 8-month follow-up after the second crown reattachment procedure.

1. Under local anesthesia with 4%articaine and epinephrine, a pulpotomy and crown reattachment procedure were performed on Tooth 11 using a rubber dam and dental microscope (Figure 1d,e). Pulpotomy was conducted under the microscope, with direct pulp capping using the bioceramic material iRoot BP Plus (Innovative BioCeramix Inc., Canada) and a base of light-cured calcium silicate TheraCal LC (Bisco Inc., United States). The bonding interface of the base tooth and fractured tooth segment was etched with 35%phosphoric acid (Heraeus Inc., Germany) and coated with 3M eighth-generation universal adhesive (3M Inc., United States). The fractured tooth segment was repositioned and bonded to the base tooth using resin of Beautifil Flow Plus F00 (Shofu Inc., Japan) and completely repositioned under the microscope. The labial bonding interface was prepared with a shallow concave short bevel for composite resin restoration using Brilliant NG (Coltene Inc., Switzerland), adjusted for occlusion, and polished.
2. One month following the reattachment of the dental fragment, a subsequent periapical radiograph revealed a calcified bridge formation at the root canal orifice of Tooth 11, with no signs of abnormality in the periapical region. At the 6-month postoperative mark, the electric pulp vitality test indicated a value of 21 for Tooth 11, while Control Tooth 21 exhibited a vitality test value of 18. The periapical radiograph continued to display the calcified bridge formation at the root canal orifice of Tooth 11, and no significant abnormalities were observed in the periapical area (Figures 1f, 1g, and 1h).
3. Sixteen months subsequent to the initial fragment reattachment procedure, Tooth 11 sustained a further traumatic fracture. Following the repositioning of the fractured crown of Tooth 11, a fluid resin was employed to temporarily secure the fractured segment on the labial aspect. A resin wing was then fabricated, and the fragment reattachment was finalized using the resin wing. The postoperative periapical radiograph indicated that the fractured crown of Tooth 11 was well positioned, with evidence of a calcified bridge image change at the root canal orifice and no significant abnormalities detected in the periapical region.
4. Eight months subsequent to the second fragment reattachment procedure and 24 months following the pulpotomy, a follow-up examination disclosed that Tooth 11 remained intact with healthy periodontal tissues. The pulp electric vitality test yielded values of 21, 21, and 20, respectively. In parallel, Control Tooth 21 exhibited electric vitality test values of 19, 18, and 18. The periapical radiograph indicated no significant abnormalities in the apical region. (Figures 2a, 2b, 2c, 2d, 2e, 2f, 2g, 2h, 2i, 2j, 2k, 2l, 2m, 2n, 2o, 2p, 2q, 2r, 2s, 2t, 2u, and 2v).

## 2. Discussion and Conclusion

Anterior tooth crown fractures are commonly treated using restorative techniques such as direct composite resin restoration, indirect restorations (e.g., full crowns and veneers), and fragment reattachment. Fragment reattachment involves adhering a salvageable fractured segment to the tooth using adhesive materials [11–13]. This method offers advantages over alternative techniques, including minimal tooth preparation, optimal esthetics, and precise color, form, and texture matching with adjacent teeth [14–16]. Additionally, fragment reattachment typically requires less chair time and is more cost-effective, reducing overall treatment costs [17–19].

In the present case, the patient sustained a crown fracture with pulp exposure. Pulpotomy was performed to preserve pulp vitality, followed by fragment reattachment using a modified resin wing technique. This approach maximized the retention of natural tooth structure [20–24]. While fragment reattachment alone might suffice for fractures without pulp involvement, pulpotomy was indicated here due to the pulp exposure, which posed a risk of infection and necrosis if left untreated.

Notably, the resin wing technique significantly enhanced repositioning accuracy during fragment reattachment. The technique entailed the following steps:

1. The fractured segment was temporarily aligned using fluid resin on the labial surface.
2. A resin wing was fabricated at the incisal edge to engage the adjacent tooth.
3. After curing, the labial resin was removed, and the bonding surfaces were prepared.
4. During reattachment, pressure was applied to the resin wing to ensure optimal seating.
5. Excess resin was eliminated, and the wing was removed after final curing.
6. A shallow concave bevel was created on the labial surface to improve restoration stability and load-bearing capacity [9, 25–27].

However, this technique may be limited by anatomical constraints, for instance, as functional support of adjacent teeth could impede resin wing fabrication and seating. Both functional and esthetic outcomes were highly satisfactory to the patient and family [28, 29]. At > 24-month follow-up, the tooth exhibited normal pulp vitality and periodontal health, though long-term monitoring remains necessary [30–35].

Pulp preservation combined with fragment reattachment represents an optimal approach for managing complex crown fractures in young permanent teeth. The resin wing technique improved repositioning precision and reduced technical sensitivity, enabling efficient case completion. Nevertheless, case selection—considering factors such as functional support of adjacent teeth—is critical for technique applicability.

## Data Availability

Data supporting this research article are available from the corresponding author or first author upon reasonable request.

## References

[1] Lauridsen E., Hermann N. V., Gerds T. A., Kreiborg S., Andreasen J. O. (2012). Pattern of Traumatic Dental Injuries in the Permanent Dentition Among Children, Adolescents, and Adults. *Dental Traumatology*.

[2] Andreasen J. O. (1970). Etiology and Pathogenesis of Traumatic Dental Injuries. A Clinical Study of 1,298 Cases. *Scandinavian Journal of Dental Research*.

[3] Andreasen F. M., Daugaard-Jensen J., Munksgaard E. C. (1991). Reinforcement of Bonded Crown Fractured Incisors With Porcelain Veneers. *Endodontics & Dental Traumatology*.

[4] Eden E., Taviloğlu E. (2016). Restoring Crown Fractures by Direct Composite Layering Using Transparent Strip Crowns. *Dental Traumatology*.

[5] Bourguignon C., Cohenca N., Lauridsen E. (2020). International Association of Dental Traumatology Guidelines for the Management of Traumatic Dental Injuries: 1. Fractures and Luxations. *Dental Traumatology*.

[6] Paulina D. P., Jain N. (2025). Treatment Modalities of Uncomplicated Crown Fracture in Anterior Maxillary Permanent Teeth: A Systematic Review. *Journal of Esthetic and Restorative Dentistry*.

[7] Marinčák D., Doležel V., Přibyl M. (2021). Conservative Treatment of Complicated Crown Fracture and Crown-Root Fracture of Young Permanent Incisor—A Case Report With 24-Month Follow-Up. *Children*.

[8] Liu H., Zhang X., Zhang X., Shi Y., Chen J., Liu X. (2024). Single- Versus Multi-Visit Approach for Fragment Reattachment in Complicated Crown-Root Fractures: A Cohort Study. *BMC Oral Health*.

[9] de Sousa A. P. B. R., França K., de Lucas Rezende L. V. M. (2018). In Vitro Tooth Reattachment Techniques: A Systematic Review. *Dental Traumatology*.

[10] Macedo G. V., Diaz P. I., De O Fernandes C. A., Ritter A. V. (2008). Reattachment of Anterior Teeth Fragments: A Conservative Approach. *Journal of Esthetic and Restorative Dentistry*.

[11] Sarapultseva M., Sarapultsev A. (2019). Long-Term Results of Crown Fragment Reattachment Techniques for Fractured Anterior Teeth: A Retrospective Case-Control Study. *Journal of Esthetic and Restorative Dentistry*.

[12] Martins A. V., Albuquerque R. C., Lanza L. D. (2018). Conservative Treatment of a Complicated Crown-Root Fracture Using Adhesive Fragment Reattachment and Composite Resin Restoration: Two Year Follow-Up. *Operative Dentistry*.

[13] Panchal D. (2019). A Case Report of Uncomplicated Crown Fracture: Tooth Fragment Reattachment. *British Dental Journal*.

[14] Khandelwal P., Srinivasan S., Arul B., Natanasabapathy V. (2021). Fragment Reattachment After Complicated Crown-Root Fractures of Anterior Teeth: A Systematic Review. *Dental Traumatology*.

[15] Lempel E., Lovász B. V., Meszarics R., Jeges S., Tóth Á., Szalma J. (2017). Direct Resin Composite Restorations for Fractured Maxillary Teeth and Diastema Closure: A 7 Years Retrospective Evaluation of Survival and Influencing Factors. *Dental Materials*.

[16] Mendes L., Laxe L., Passos L. (2017). Ten-Year Follow-Up of a Fragment Reattachment to an Anterior Tooth: A Conservative Approach. *Case Reports in Dentistry*.

[17] Pinheiro E. S., Almeida J. C. F., Garcia F. C. P. (2024). An Assessment of Brazilian Dentists’ Knowledge About Tooth Fragment Reattachment: A Cross-Sectional Study. *Dental Traumatology*.

[18] Glendor U. (2000). On Dental Trauma in Children and Adolescents. Incidence, Risk, Treatment, Time and Costs. *Swedish Dental Journal. Supplement*.

[19] Bissinger R., Müller D. D., Hickel R., Kühnisch J. (2021). Survival Analysis of Adhesive Reattachments in Permanent Teeth With Crown Fractures After Dental Trauma. *Dental Traumatology*.

[20] Bimstein E., Rotstein I. (2016). Cvek Pulpotomy - Revisited. *Dental Traumatology*.

[21] Wang G., Wang C., Qin M. (2017). Pulp Prognosis Following Conservative Pulp Treatment in Teeth With Complicated Crown Fractures-a Retrospective Study. *Dental Traumatology*.

[22] Rao Q., Kuang J., Mao C. (2020). Comparison of iRoot BP Plus and Calcium Hydroxide as Pulpotomy Materials in Permanent Incisors With Complicated Crown Fractures: A Retrospective Study. *Journal of Endodontics*.

[23] Matoug-Elwerfelli M., ElSheshtawy A. S., Duggal M., Tong H. J., Nazzal H. (2022). Vital Pulp Treatment for Traumatized Permanent Teeth: A Systematic Review. *International Endodontic Journal*.

[24] Donnelly A., Foschi F., McCabe P., Duncan H. F. (2022). Pulpotomy for Treatment of Complicated Crown Fractures in Permanent Teeth: A Systematic Review. *International Endodontic Journal*.

[25] Ghoreishizadeh A., Mohammadi F., Rezayi Y., Ghavimi M., Pourlak T. (2020). Comparison of Shear Bond Strengths With Different Bevel Preparations for the Reattachment of Fractured Fragments of Maxillary Central Incisors. *Dental Traumatology*.

[26] Chazine M., Sedda M., Ounsi H. F., Paragliola R., Ferrari M., Grandini S. (2011). Evaluation of the Fracture Resistance of Reattached Incisal Fragments Using Different Materials and Techniques. *Dental Traumatology*.

[27] Chandran R., Rayar S., Ravi A. B., Haridas K. (2020). Comparative Evaluation of Fracture Resistance of Incisor Fragments Using Simple, Bevel, Internal Groove Preparation Designs and Reattached With Nanocomposites: An in Vitro Study. *Journal of Pharmacy & Bioallied Sciences*.

[28] Shahmohammadi R., Sheikhnezami M., Moradi S., Jafarzadeh H., Azarpazhooh A. (2021). Treatment Outcomes of Permanent Immature Teeth With Crown Fracture: A Retrospective Cohort Study. *Journal of Endodontics*.

[29] Andreasen F. M., Kahler B. (2015). Pulpal Response After Acute Dental Injury in the Permanent Dentition: Clinical Implications—A Review. *Journal of Endodontics*.

[30] Garcia F. C. P., Poubel D. L. N., Almeida J. C. F. (2018). Tooth Fragment Reattachment Techniques-a Systematic Review. *Dental Traumatology*.

[31] Abbott P. V. (2023). Indications for Root Canal Treatment Following Traumatic Dental Injuries to Permanent Teeth. *Australian Dental Journal*.

[32] Sharif M. O., Tejani-Sharif A., Kenny K., Day P. F. (2015). A Systematic Review of Outcome Measures Used in Clinical Trials of Treatment Interventions Following Traumatic Dental Injuries. *Dental Traumatology*.

[33] Soliman S., Lang L. M., Hahn B. (2020). Long-Term Outcome of Adhesive Fragment Reattachment in Crown-Root Fractured Teeth. *Dental Traumatology*.

[34] Galler K. M., Grätz E. M., Widbiller M., Buchalla W., Knüttel H. (2021). Pathophysiological Mechanisms of Root Resorption After Dental Trauma: A Systematic Scoping Review. *BMC Oral Health*.

[35] Bissinger R., Müller D. D., Reymus M. (2021). Treatment Outcomes After Uncomplicated and Complicated Crown Fractures in Permanent Teeth. *Clinical Oral Investigations*.

